# Testing the effects of perceptual grouping on visual search in older adults

**DOI:** 10.1038/s41598-022-23139-3

**Published:** 2022-11-10

**Authors:** Li Jingling, Sung-Nan Lai

**Affiliations:** Graduate Institute of Biomedical Sciences, China Medical University, No. 91, Xue-Shi Road, Taichung, 40402 Taiwan

**Keywords:** Psychology, Human behaviour

## Abstract

Visual search is to find targets while ignoring distractors. Previous studies established that a target is more difficult to identify if aligned collinearly with other items, called the collinear search impairment. Since older adults have lower perceptual grouping ability than younger adults, benefits in visual search may occur for older adults for they may be less distracted by the collinear distractors. Three experiments were carried out to compare 45 younger and 45 older healthy adults. Participants were asked to identify a local target either in the column with items collinearly aligned to each other (the overlapping condition) or in the background (the non-overlapping condition), and the response difference between the two conditions is the collinear search impairment. Results showed that both groups showed reliable search impairment specific to collinear distractor regardless of grouping difficulty and task demands, and the impairment strength increased with the grouping strength of the collinear distractor. Further analysis revealed that the response times of older adults increased in a multiplicative manner to that of younger adults, suggesting that longer response of older adults spread to multiple underlying processing including grouping and suppression of collinear distractors. Together, the results suggest that older adults were still distracted in visual search even when grouping was required on a distractor. Our findings also highlight how general slowing may delay suppression processing in visual search.

Efficient visual search requires rapid updating of a target location while ignoring distractors^1^, which is essential for many cognitive operations. Visual search occurs frequently without notice in daily life because it usually finishes within seconds. For instance, a series of searches during breakfast could include a piece of bread, a butter knife, and a piece of butter, while ignoring a vase of flowers on the table. The ability to ignore distractors is associated with cognitive inhibition and working memory, which declines in older adults, leading to less efficient exclusion of distractors^2,3^. This study hypothesized that such stronger distraction might become weaker if the distractor requires perceptual grouping, which is an ability evidently declined in older adults^4–9^. If this hypothesis holds true, declined perceptual ability with age in some situations could be positive and even contributes to a better cognitive operation for older adults.

The current study focused on the collinear search impairment, which was an interference induced by a globally grouped distractor to the local targets in visual search^10–17^. In our previous study, collinear search impairment was demonstrated when a local target overlapped with a global collinear structure (Fig. 1A); an impairment compared to the condition where the target was in the background. Figure 1 shows an example of the search display demonstrating the effect. The display was filled with horizontal bars except that one column of the bars in the search display was vertical and aligned head-to-tail, called the collinear distractor (Fig. 1A). The target (Fig. 1C) was a black left or right tilted segment in the middle of a white bar. Participants were asked to discriminate whether the target was tilted leftward or rightward in each trial. The target was designed to be in the column of the collinear distractor by chance, making the distractor task-irrelevant. Since the collinear distractor is salient and well-grouped^18^, the overlapping target should enjoy the salience and capture attention^19–21^. Interestingly, the opposite was observed: responses were faster in the non-overlap condition. Such search impairments were not observed in a similar design where the global structure was changed to a non-collinear distractor^13^ (Fig. 1B), suggesting the importance of collinear grouping in search impairment.

**Figure 1:**
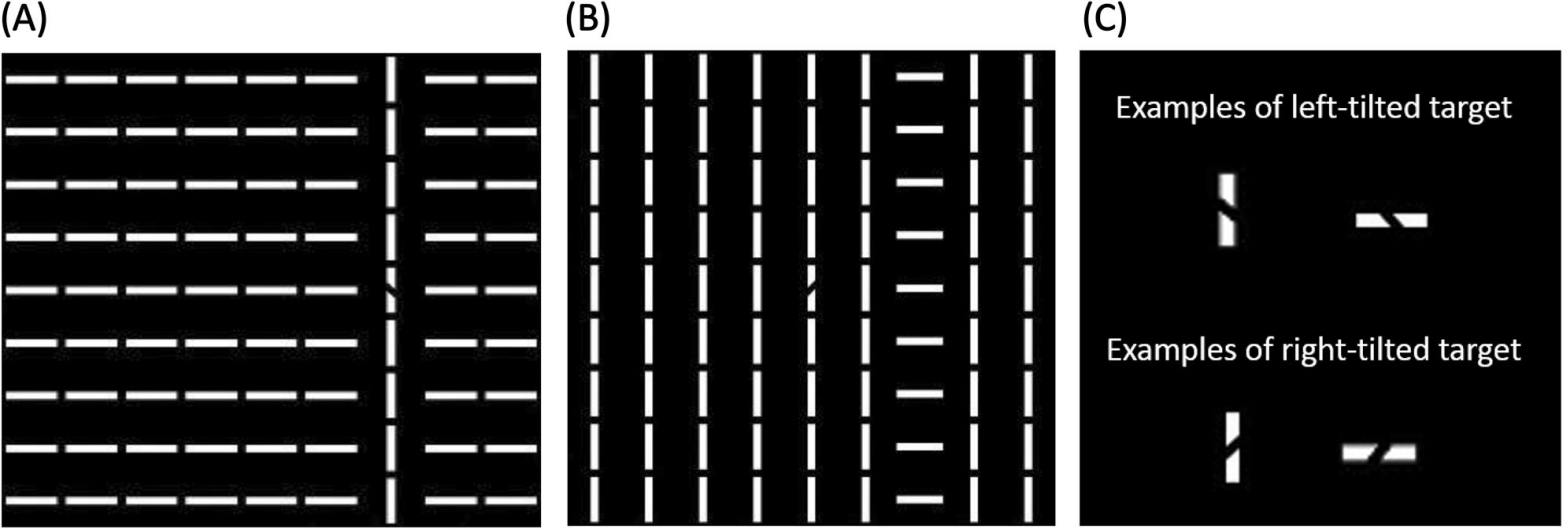
Examples of the search display used in Experiment 1. (**A**) The overlap condition with a collinear distractor: the display contains a collinear distractor (vertical white lines), and the target (i.e., the black leftward-tilted segment) is in the column of the collinear distractor. (**B**) The non-overlap condition with a non-collinear distractor: the display contains a non-collinear distractor, where the black rightward-tilted target is not in the distractor column (horizontal white lines). (**C**) Examples of possible targets in the search display. Regardless of the orientation of the bar, the upper panel presents the left-tilted targets, and the lower panel presents the right-tilted targets.

The reasons why a collinear distractor impairs visual search remain unclear. Relative salience between the target and the distractor^11,15^, attentional control setting on task demands^11,17^, and attentional focus size^10^ could not alter such search impairment. The critical processing for the collinear search impairment was inferred to be at or beyond neural computations in the early visual cortex^16,22^. Nevertheless, the collinear search impairment is associated with the grouping strength of the collinear distractor: the longer the collinear distractor, the stronger the observed impairment^13^. Furthermore, curving the collinear distractor could still generate search impairment^15^, suggesting the critical factor is not the law of similarity (i.e., grouped by the same features, such as the non-collinear case, Fig. 1B), but the law of good continuity (i.e., smooth alignment as the collinear case, Fig. 1A). Finally, search impairment decreased with the enlargement of the scale of the elements (i.e., decreasing grouping strength of the distractor^14^). In summary, search impairment occurs when the target is on a collinearly grouped distractor.

Compared with younger adults, older adults were shown to have greater difficulty in collinear grouping. Del Viva and Agostini^6^ found that older adults had lower sensitivity than did younger adults in locating a collinear contour among distracters. Roudaia et al.^4^ showed that older adults did not exhibit the advantages that younger adults had, such as having a lower threshold for detecting a collinear contour (i.e., with elements arranged parallel to the contour path) than a non-collinear contour (i.e., with elements radial to the path). Older adults also took longer to discriminate the shapes of a collinear contour embedded in distractors^5^. McKendrick et al.^9^ found that older adults required more elements than did younger adults to reach the same accuracy in a discrimination task on the shape of contours. Such differences in grouping ability between ages were more profound in more challenging groupings, such as a contour path that was not straight or in a heterogeneous background^5,7,8^. Thus, older adults take longer and require more clues to integrate collinear contours, indicating a decline in collinear grouping ability.

Although the decreased grouping ability of older adults is considered a disadvantage, this age-related decline could be beneficial in situations where grouped distractors prolong or impair visual search. The collinear search impairment occurs only when the distractor was grouped collinearly^13,14^. If collinear grouping is decreased in older adults^4–6,8,9^, then a collinear distractor may be less distracting to older adults than to younger adults. Older adults may also perceive different search displays equally (e.g., Fig. 1A,B)^4^, regardless of how the distractor is grouped. Therefore, older adults might be immune to collinear search impairment, or are less affected by collinear distractors. If so, older adults could exhibit more efficient searches than younger adults when a target is embedded in a collinear structure.

To the best of our knowledge, this is the first study to focus on the effect of collinear grouping on the distractors in visual search for older adults. The most reliable observation from previous studies on the effect of age in visual search was general slowing^2,23–28^. It is thus possible that older adults might respond slower, but they are not susceptible to collinear search impairment. Three experiments were designed to investigate whether older adults would be affected by collinear distractors as younger adults in visual search. In experiment 1, the classic search display with collinear (Fig. 1A) and non-collinear (Fig. 1B) distractors was used. In experiment 2, the grouping strength of the collinear distractors was manipulated. In experiment 3, the collinear distractor was curved to increase grouping difficulty. Finally, the performance of younger and older adults in three experiments was fit into a Brinley plot and a state trace for meta-analysis^29,30^. The Brinley plot provided clues on whether search impairment for the two groups was attributed to the same underlying mechanisms, and the state trace identified whether older adults took longer to respond than did younger adults due to the simple addition of processing stages (e.g., compensation processing) or inflating slower processing in multiple stages (e.g., maintenance processing^31^). Together, the results could provide evidence for whether the declined grouping ability of older adults could be beneficial in visual search.

## Methods

### Participants

Power analysis by G*Power 3.1.9.4 using repeated measures, within-between interaction ANOVA for two groups, two number of measures, and a η_p_^2^ of 0.29 (from Ref.^14^ which used the same element size as the current study) estimated that the number of required participants was 10 to reach the following levels: alpha 0.05, power 0.90, and correlation among repeated measurements 0.5. The number of participants was increased in each experiment due to the inclusion of older adults, who are known for large variances in responses^30,32^. The inclusion criteria for older adults were the following: (1) no brain injury or history of brain surgery; (2) normal or corrected-to-normal vision, tested with a Sloan E chart passing visual acuity 0.8; and (3) a Montreal Cognitive Assessment (MoCA) score of higher than 26, which is considered normal cognitive ability^33^. All methods were performed in accordance with the Declaration of Helsinki. All participants signed the informed consent approved by the Research Ethics Committee of China Medical University Hospital (CMUH105-REC2-124). None of them repeated in experiments. They were unaware of the goal of the study in advance, and received NT$100 (NT$200 for experiment 3) as compensation for their time.

There were 15 younger adults from China Medical University (mean age 23.8 years; range 20–30 years; 3 males) and 17 older adults from the Stella Matutina Social Welfare Foundation (mean age 74.1 years; range 65–75 years; 2 males) in experiment 1. Two of the older adults did not finish the entire task due to an interruption by personal events; thus, only 15 results from the older adults were analyzed. Another 12 young people at China Medical University (mean age 22.7 years; range 20–30 years; 4 males) and 14 older adults who performed their morning exercises in Taichung Beitun Children Park (mean age 71.9 years; range 65–75 years; 3 males) were recruited for experiment 2. Data from two older adults were excluded: one responded too slowly (mean RT = 11,608 ms) and the other made too many errors (accuracy = 67.82%). Thus, data from 12 younger adults and 12 older adults were included in experiment 2. In experiment 3, because heterogeneous search displays^15^ were adopted, the sample size was increased in both age groups. Eighteen older adults (mean age 74.57 years; range 65–83 years; six males) from the Fo Guang Shan Open University Taichung Campus and 18 younger adults (mean age 22.67 years; range 20–29 years; six males) from online advertisements were recruited. The data of all the participants were used for further analysis in experiment 3.

### Apparatus and stimuli

The experiment was carried out in a dimly lit room and tested individually. The participants sat, and with a chinrest to help stabilize the head, viewed the stimuli shown on a 19″ LED monitor (ASUS) from approximately 60 cm. Two numbered keypads, on which two specific numbers were marked, were installed separately on the right-hand and left-hand sides of the monitor to serve as input devices. The stimuli were presented and the data were recorded using E-prime 2.0 Professional.

Figure 1 shows the examples of the search display in experiment 1. The display with a collinear distractor was formed by a grid of 9-by-9 white horizontal bars except for one column of vertical bars (Fig. 1A), and the display with a non-collinear distractor reversed the orientation of the bars (Fig. 1B). The unique column in both displays was the distractor. All stimuli were displayed on a black background. The visual angle of each bar was extended to 2.08° × 0.29°. A fixation cross was presented with a visual angle of 2.08° × 2.08° on a blank display, serving as the fixation display shown between trials. The target was a tilted left or right line in one of the bars (Fig. 1C). The luminance level was 197 cd/m^2^ for white and 1.08 cd/m^2^ for black. The distractors were randomly located in the third, fifth, or seventh column of the display (Fig. 1). The target was always located at the fifth row (the central row) and was randomly moved between the same three columns. In experiment 2, the search display was 13 × 13 bars to enable more freedom in manipulating the distractor length (Fig. 4A). The possible target and distractor positions were modified to be at the 5th, 7th, and 9th column to be aligned to the center of the display.

In experiment 3, the display grid size was 13 × 17 bars to provide more freedom in the localization task (Fig. 5A). The possible target and the distractor locations were the 5th, 8th, 10th, and 13th columns. In the curved condition (Fig. 5A left two panels), the collinear distractor was composed of 11 bars in a smooth orientation that formed either an ‘S’ or ‘Z’ shape. Other bars were pseudo-randomly oriented such that two adjacent bars were never in the same orientation. Totally 8 possible orientations were used in the background. The center bar (the fifth bar) of the distractor was kept vertical and was the location to present the target in the overlap condition. In the straight condition (Fig. 5A right two panels), the same layout as the experiments 1 and 2 were used.

### Design

The three experiments were three-factor mixed designs. The between factor was age group, and the within factors were the distractor type (collinear or non-collinear in experiment 1, distractor length in experiment 2, and curved or straight in experiment 3) and target type (overlap or non-overlap). An overlap target was the condition when the target was located on the column of the distractor (e.g., Fig. 1A), otherwise were non-overlap targets (e.g., Fig. 1B). To ensure that the distractor was task-irrelevant^19–21^, the locations of the target and the distractor were varied independently so that the participants could not predict the location of the target from the distractor. The probability of overlap targets was one-third in experiment 1 and 2 and one-fourth in experiment 3, respectively, because there were three possible locations in the former and four in the latter. In experiment 1, overlap and non-overlap conditions respectively generated 36 and 72 trials for the collinear condition; and another 36 and 72 trials for the non-collinear condition, leading to a total of 216 trials. In experiment 2 (Fig. 4A), the distractor length could be 3, 5, 9, or 13 bars. Each length condition had 18 overlap trials and 36 non-overlap trials, comprising a total of 216 trials. In experiment 3 (Fig. 5A), there was 40 overlap and 120 non-overlap trials for the straight condition, and another 40 and 120 trials for the curved condition, resulting 320 trials in total. The trials were presented in a completely randomized sequence.

### Procedures

In experiments 1 and 2, each trial began with a fixation cross for 500 ms, followed by the search display until participants responded. A blank screen was then displayed for 500 ms before the next trial. Participants were asked to identify the tilted black line on one of the bars (the target, Fig. 1C) and answer ‘left’ or ‘right’ by pressing the corresponding key on the number keypad. Six practice trials were performed before the actual experiment. Participants were encouraged to respond as quickly as possible while maintaining accuracy. There was no feedback. During the experiment, two breaks were provided. The experiment took approximately 15 min to complete.

In experiment 3, the task was to determine whether the target was on the left or right side of the search display (Fig. 5A), regardless of its orientation. If the target was at the left side of the screen, the correct answer was to press the left key, and vice versa. Participants completed ten practices before data collection. There were four breaks during experiments. All other details were the same as those of experiment 1.

### Statistics and cross-experiment analysis

RT and accuracy were recorded in three experiments. To avoid outliers, responses with RT longer than two SDs of the mean for that individual were discarded. Only correct responses were included in RT analysis. Since older adults usually required more trials to stabilized their response, we excluded first 30 trials in the formal experiment for further analysis for both age groups. In consideration of possible variations in response times (RTs) between age groups, RTs of individual were transformed into z scores (RTz). RTs of each trial were transformed to RTz, where the RT of each trial was subtracted from each individual’s mean RTs and then divided by the SD of the individual. A positive and negative RTz respectively indicates an RT longer and shorter than the individual mean. The data for RTz and accuracy were analyzed using a three-way mixed analysis of variance (ANOVA) for the factors age group (younger and older adults), distractor type (varied according to experiments), and target type (overlap or non-overlap).

Interpretations based on age group comparisons should be made with caution because RT differences between conditions may also enlarged with general slowing^30,34^. This is because longer RTs for a more difficult condition might become even longer if the individual had a slower processing speed. Z transformation of RTs could eliminate individual difference quite well; however, if participants showed variability across conditions (i.e., different processing rates), z value would exaggerate for those with longer RTs^35^. To understand more details on RT differences between age group and conditions, the Brinley plot and state-space analysis^29,30^ were applied to compare the performance of two age groups across three experiments.

**Figure 2:**
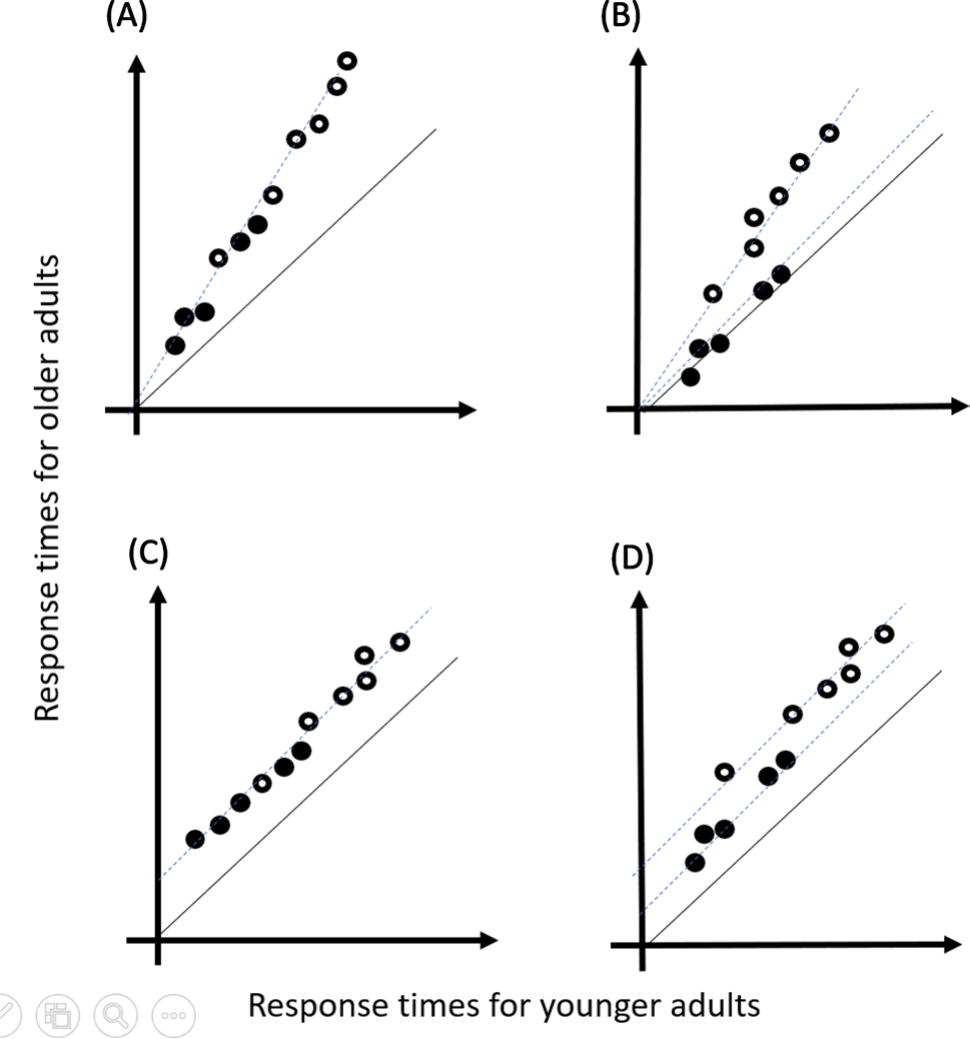
The hypothetic plots for possible results of the Brinley plot. The hypothetic data for the non-overlap condition (the easier condition) were marked with black dots and that for overlap condition (the more difficult condition) were white dots. The diagonals of the plot were marked with black while the fitted regression lines were marked with dotted blue lines. (**A**) Multiplicative complexity, equivalent processing speed for both conditions. (**B**) Multiplicative complexity, less efficient processing for overlap than non-overlap conditions. (**C**) additive complexity, equivalent processing stages, and (**D**) additive complexity, some additional processing stages for overlap than non-overlap conditions.

The Brinley plot can help us understand whether the more difficult overlap condition required more processing duration than the non-overlap condition at the critical processing stage (the additive complexity) or whether the delayed processing for the overlap condition was inflated into several steps in the processing chain compared to the non-overlap condition (the multiplicative complexity)^29,30,32,36^. In the Brinley plot, the horizontal dimension was RT scales from younger adults, and vertical dimension was that from older adults. Figure 2 illustrates four possible consequences in comparing overlap and non-overlap RTs for older and younger adults in the Brinley plot. In Fig. 2, RTs for easier conditions (i.e., non-overlap condition) were plotted with black dots and that for harder (i.e., overlap condition) were white dots. It is assumed that processing speed for a harder condition (e.g., overlap) can be predicted via either a multiplicative (i.e., a fixed ratio, Figs. 2A,B) or an additive (i.e., increased with a constant, Figs. 2C,D) manner from an easier condition (e.g., non-overlap). The multiplicative complex is that the processing speed for older adults is delayed in many stages in the more difficult condition than younger adults, thus would lead to a larger slope (deviating from the diagonal) in the Brinley plot (Fig. 2A). If the processing speed delayed in different ratio in the two difficulty levels, two rather than one regression lines for these two conditions would be needed to fit the data (Fig. 2B). If older adults required some extra stages than younger adults, then the fitted regression line would have a positive intercept in the Brinley plot (Fig. 2C). If overlap condition required an additional processing than non-overlap condition, extra intercept is expected and two separate regression lines are needed to account for the data (Fig. 2D). An *R*^2^ of the linear regression larger than 0.90 can be considered a good fit^36^. Real data might combine the two complexities and the fitted regression lines might have a positive intercept and a slope larger than one.

On the other hand, the state trace from the state space can help us understand whether older adults need to have more processing stages than younger adults in the visual search (the additive complexity) or whether longer RTs for the older adults were due to inflation into several steps in the processing chain compared to younger adults (the multiplicative complexity). The state space was generated by plotting data from the non-overlap condition in the horizontal dimension and the overlap condition in the vertical dimension. The illustration of the four possible results in Fig. 2 also applies to the state space by replacing the labels of the dimensions. A single regression line fitting data from the younger and older adults means that the same underlying mechanism can be inferred for both age groups (Fig. 2A or C); however, two regression lines for the two age groups mean that older adults adopt a different manner to conduct search impairment (Fig. 2B or D). Furthermore, the slope of the regression line of the state trace provides clues of how older and younger adults differ. Specifically, a regression line parallel to the diagonal is considered additive complexity (Fig. 2C or D), implying that older adults requires an additive stage of processing or a prolonged stage. A fitting line with a greater slope than the diagonal is considered a multiplicative complexity (Fig. 2A or B), implying that several processing stages are altered or processing speeds of older adults are increased as a ratio to that of younger adults^29^.

To do the Brinley plot and state trace, the grand mean of RT in each condition in each age group was first extracted. Data from Experiment 1 in the non-collinear distractor condition were excluded for it was not a condition expected to generate search impairment^12,13^. In total, seven conditions (the collinear distractor condition in experiment 1, four distractor length in experiment 2, and the straight distractor and the curved distractor conditions in Experiment 3) were calculated for each age group. The data for the overlap and non-overlap were separately recorded, leading to 14 points for each age group.

## Results

Screening of RT with two SDs of the mean for each participant resulted in the removal of 3.66% and 3.85% in experiment 1, 3.39% and 2.08% in experiment 2, and 5.22% and 2.56% in experiment 3, for older and younger adults, respectively. The de-identified raw data for each experiment was shown in supplementary material.

### Experiment 1: Only collinear grouping induced search impairment

**Figure 3:**
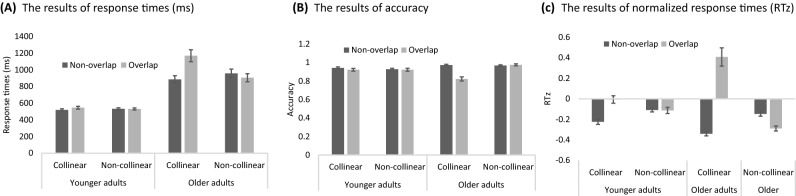
Results of Experiment 1: Reaction times of younger and older adults under collinear and non-collinear conditions. Error bars represent the standard error of the mean.

Figure 3 shows the results in experiment 1. The 3-way mixed ANOVA on RT (Fig. 3A) revealed significant main effects of age group, distractor types, and target types, *F*(1,28) = 69.80, 19.86, and 41.18, η_p_^2^ = 0.71, 0.30, and 0.43, *p*s < 0.001, respectively. The two-way and three-way interactions were all significant (more details of ANOVA please see Table S1 in the supplementary materials). Interestingly, older adults showed collinear search impairment in the collinear distractor condition, *F*(1,56) = 163.85, η_p_^2^ = 0.72, *p* < 0.001, and non-collinear attentional capture, *F*(1,56) = 5.34, η_p_^2^ = 0.02, *p* = 0.025. The RT effects for younger adults were not significant.

The 3-way ANOVA on accuracy (Fig. 3B) also showed significant main effects on distractor types, and target types, *F*(1,28) = 17.40 and 25.12, η_p_^2^ = 0.25 and 0.38, *p*s < 0.001, but not significant for age group, *p* = 0.566. The two-way and three-way interactions were all significant. The older adults showed collinear search impairment in the collinear distractor condition, *F*(1,56) = 68.14, η_p_^2^ = 0.54, *p* < 0.001.

The 3-way ANOVA on RTz (Fig. 3C) showed significant main effects on distractor type and target type, *F*(1,28) = 18.30 and 59.15, η_p_^2^ = 0.28 and 0.59, *p*s < 0.001, but not significant for age group, *p* = 0.29. The two-way and three-way interactions were all significant. Both groups showed the collinear search impairment in the collinear distractor condition, *F*(1,56) = 144.60 and 12.27, η_p_^2^ = 0.66 and 0.06, *p*s < 0.001, for older adults and younger adults, respectively. Only older adults showed attentional capture in the non-collinear condition, *F*(1,56) = 5.21, η_p_^2^ = 0.02, *p* = 0.026. Younger adults did not show reliable attentional capture effect, p = 0.953.

### Experiment 2: Search impairment increased with collinear grouping strength

**Figure 4:**
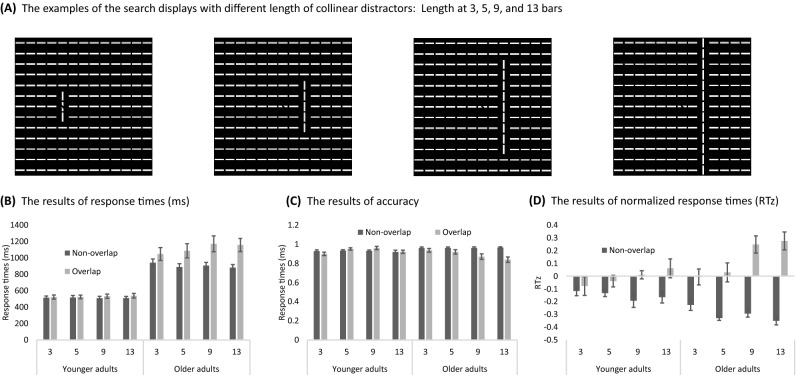
The examples of four distractor length conditions (**A**) and the results of younger and older adults in each condition in response times (**B**), accuracy (**C**), and normalized response times (**D**) in experiment 2. The length of the collinear distractor was 3-bar, 5-bar, 9-bar, and 13-bar. The target could overlap with the collinear distractor (the 3-bar case) or be non-overlapped (the 5-, 9-, and 13-bar cases). (**B**) Response times, accuracy (**C**), and normalized response time (RTz, **D**) for the younger and older adults are plotted according to distractor length and target type (overlap or non-overlap). Error bars represent the standard error of the mean.

Figures 4 show the results of experiment 2 for younger and older adults, respectively, and Table S2 shows the details of ANOVA. The three-way mixed ANOVA on RT (Fig. 4B) revealed a significant main effect on age group, target type, and distractor length, *F*(1,22) = 58.17, 18.16, and 4.43, η_p_^2^ = 0.73, 0.34, and, 0.15, *p*s < 0.001, respectively. The two-way and three-way interactions were all significant. For older adults, RT under the overlap condition was significantly longer than non-overlap condition in all distractor lengths, *F*(1,88) = 6.47, 22.79, 40.84, and 43.54, η_p_^2^ = 0.03, 0.11, 0.20, and, 0.21, *p*s < 0.001, for length 3-, 5-, 9-, and 13-bar, respectively. The differences were not statistically significant for younger adults.

ANOVA on accuracy (Fig. 4C) showed significant main effect of target type, *F*(1,22) = 12.37, η_p_^2^ = 0.24, *p* = 0.002, showing that the collinear search impairment can be observed in accuracy. Other main effects were not significant. The two-way interaction of age group and target type was significant, *F*(1,22) = 16.78, η_p_^2^ = 0.33, *p* < 0.001, as well as the interaction of age group and distractor length, *F*(1,22) = 3.02, η_p_^2^ = 0.11, *p* = 0.036. The three-way interaction was also found, *F*(3,66) = 3.49, η_p_^2^ = 0.13, *p* = 0.020. Detailed analysis showed that the collinear search impairment was observed for distractor length 9-bar and 13-bar conditions for the older adults, *F*(1,88) = 13.87 and 27.42, η_p_^2^ = 0.10 and 0.20, *p*s < 0.001, respectively.

Figure 4D showed the results of RTz. The ANOVA showed that the main effect of target type was significant, *F*(1,22) = 41.30, η_p_^2^ = 0.56, *p* < 0.001. The two-way interaction of age group and target type was found, *F*(1,22) = 10.84, η_p_^2^ = 0.15, *p* = 0.003, suggesting that the collinear search impairment was larger for older adults than younger adults. The two-way interaction of age group and distractor length was also found, *F*(1,22) = 9.67, η_p_^2^ = 0.29, *p* < 0.001. Therefore,, the prolonged RT with distractor length was more profound in the older adults than younger adults.

To understand whether the size of search impairment increased with distractor length, a two-way mixed ANOVA was performed for RTz differences between the overlap and non-overlap conditions (the size of search impairment, more details in Table S3). Results showed a significant main effect for age group, *F*(1,22) = 10.84, η_p_^2^ = 0.33, *p* = 0.003, suggesting a stronger collinear search impairment observed for older adults than younger adults. Also, significant main effect of distractor length was observed, *F*(3,66) = 9.67, η_p_^2^ = 0.29, *p* < 0.001. Thus, the longer the distractor length, the stronger the collinear search impairment was observed. The interaction was not significant, *p* = 0.333. Importantly, a significant linear trend in distractor length was observed for younger adults, *F*(1,66) = 5.91, η_p_^2^ = 0.08, *p* = 0.018; and older adults, *F*(1,66) = 25.86, η_p_^2^ = 0.28, *p* < 0.001, indicating that the search impairment increased with distractor length for both age groups.

### Experiment 3: Curved collinear distractor can still impair search

**Figure 5:**
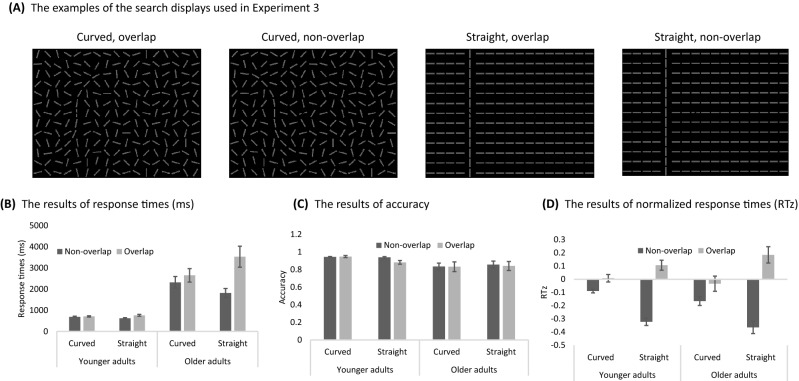
Examples of the search display in the curved and the straight conditions, and the results of younger and older adults in the conditions in experiment 3. (**A**) Examples of the displays used in experiment 3. From left to right are the curved overlap, curved non-overlap, straight overlap, and straight non-overlap conditions. (**B**) The results of response times. (**C**) the results of accuracy, and (**D**) the results of normalized response time (RTz). Error bars are the standard error of the mean.

Figures 5B shows RT results of experiment 3 for younger and older adults, respectively. More details of ANOVAs can be found in Table S4. The three-way mixed ANOVA showed a main effect of target type, *F* (1, 34) = 17.09, η_p_^2^ = 0.27, *p* < 0.001, indicating that search impairment was observed regardless of whether curved or straight distractors were used. A main effect of age group was also observed, *F* (1, 34) = 34.87, η_p_^2^ = 0.51, *p* < 0.001, as well as the main effect for distractor type, *F* (1, 34) = 6.17, η_p_^2^ = 0.13, *p* = 0.018. Two-way and three-way interactions were all significant.

Figure 5C shows results of accuracy. The ANOVA result showed the main effect of age group, *F* (1, 34) = 5.03, η_p_^2^ = 0.13, *p* = 0.031; the older adults made more errors than the younger adults. Other main effects were not significant. The two-way interaction of age and distractor type was found, *F* (1, 34) = 11.42, η_p_^2^ = 0.24, *p* = 0.002. Thus, the accuracy of older adults was more affected by distractor types than that of younger adults. The interaction of distractor type and target type was also found, *F* (1, 34) = 5.24, η_p_^2^ = 0.13, *p* = 0.028, suggesting the collinear search impairment was smaller for the curved than the straight condition. The three-way interaction was not significant, *p* = 0.164.

Figure 5D shows the RTz results of Experiment 3. ANOVA for RTz showed significant main effect of target type, *F* (1, 34) = 62.23, η_p_^2^ = 0.64, *p* < 0.001. Thus, regardless of curved or straight conditions for older or younger adults, the general effect of search impairment was significant. Also, the interaction between distractor type and target type was found, *F* (1, 34) = 91.72, η_p_^2^ = 0.72, *p* < 0.001. Therefore, the collinear search impairment was larger for the straight condition than curved condition. Other interactions were not significant.

### The Brinley plot and state trace

**Figure 6:**
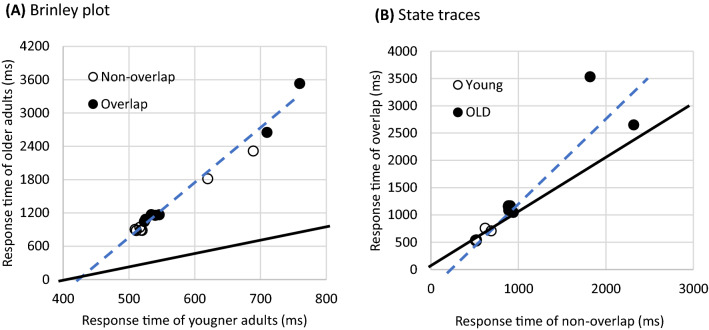
The Brinley plot (**A**) and state traces (**B**) of the three experiments. Each dot represents one experimental condition in experiments 1–3. The black line illustrates the diagonal of the plot, and the blue dashed line is the fitted linear regression.

Figure 6 illustrates the results of the meta-analysis of the three experiments. The Brinley plot (Fig. 6A) showed that the variance of the two age groups can be explained by a simple linear regression, *R*^2^ = 0.98. Separately fitting for non-overlap (*R*^2^ = 0.99) and overlap (*R*^2^ = 0.99) conditions did not considerably increase *R*^2^. The slope of the fitted regression was 9.52 (larger than 1), and the intercept was − 3989 (not a positive increment); suggesting a multiplicative complexity^29^ (Fig. 2A). Thus, a more parsimonious explanation was concluded by taking the single linear regression, in that the performance of older adults was a function of younger adults in certain expansions of RTs, from an easier to more difficult condition.

Figure 6B shows the state trace for the three experiments. The linear regression of the total data reached the highest explanation, *R*^2^ = 0.85. Separation of fitting revealed less well-fit for younger (*R*^2^ = 0.76) and older (*R*^2^ = 0.80) adults; thus, a single regression can fit the data (Fig. 2A). The slope of regression was 1.53 and the intercept was − 232.14, suggesting a multiplicative complexity for older adults in dealing with the more difficult condition (overlap condition) relative to the less difficult one (non-overlap condition). The slope suggesting that the increment of RTs of older adults was not simply an additive manner from younger adults; rather, multiple steps/processing were postponed in older adults.

## General discussion

This study examined whether older adults were as affected by task-irrelevant collinear distractors in visual search as younger adults. Results showed that older adults, like younger adults, responded slower to a target overlapped with a collinear distractor compared to a target placed in the background. This search impairment was limited to the condition where the distractor was organized collinearly (experiment 1), and increased with the length of the collinear distractor (experiment 2), and can still be observed in a condition where the distractor was curved and was embedded in a heterogenous background (experiment 3). The Brinley plot revealed that single linear regression accounted for 98% of the variance for data from both age groups, suggesting that the performance of older adults was like younger adults, though generally slower. Such difficulty–related increment in response time for older adults occurred in multiple processing stages. These findings suggest that older adults did not search for a target in an alternative manner to compensate for their less efficient perceptual grouping ability; rather, they simply completed the task slower.

Our findings show that healthy aging does not lead to alternative consequences in visual search with the exception of slowing, which is consistent with previous observations using feature or conjunction searches^2,23,25–27,32,37–40^. Our work took one step further in that slowing also applies to distractor processing. Collinear search impairment depends on collinear grouping to mask a local target^13,15^. While older adults exhibited declined grouping ability^5,7,8^, they could not take advantage of this phenomenon. Multiplication complexity from the state trace (Fig. 6B) suggests that the older adults faced extra-proportional challenges in the overlap conditions compared with the non-overlap conditions. This can be attributed to challenges from multiple levels, such as visibility of the target bar^41^, crowding effect of the complex surroundings^42^, collinear grouping and similarity grouping of the search display^4^, interference from the collinear distractor to the discrimination of target orientation^13^, and remembering the stimuli-response mapping^29,37^. Our data suggest that older adults did not omit any of these stages; rather, they used the same mechanism as younger adults did. However, the processing was prolonged. The current findings are consistent with the ‘maintenance’ account for aging^31^. The less efficient grouping ability of older adults on the collinear stimuli did not let the older adults process the search display in an alternative way, such as compensatory performance via other brain regions or resources^31^.

Our data also imply that age impact in visual search may be mainly top-down. For instance, in difficult visual search, older adults showed weaker or delayed electroencephalogram signals to targets than younger adults^3,38,39^, suggesting that older adults reduced their attentional resources to the target processing. In behavioral, this delay of attention involvement related to general slowing. Older adults also showed larger cost compared to younger adults when an illusory contour was a distractor; however, such age difference was not found when the illusory contour was the target^3^. Wiegand et al.^3^ explained that the global forms might attract attention in a higher priority than local forms, and older adults had more difficulty to suppress such default tendency. Related to our findings, the older adults may, on the one hand, have shortage of resources to discriminate the local target and, on the other hand, be less able to suppress global distractor, leading to a stronger delay in the overlapping than the non-overlapping conditions.

The fact that collinear search impairment was also observed in older adults reveals the involvement of distractor suppression in collinear search impairment. Grouping of the collinear distractor seems mandatory, regardless of personal grouping ability. The recently proposed “signal suppression hypothesis^43^” states that a salient and task-irrelevant stimulus can capture attention in a stimulus-driven manner; however, such attentional capture can be suppressed before the initial shift of attention^44,45^. Although studies of the signal suppression hypothesis usually compared the presence and the absence of a salient distractor^46,47^, rather than the overlap and non-overlap of the target and the distractor as in the case of current study, such distractor suppression increased when the salient distractor was spatially closer to the target^48^. The overlap condition had a zero distance between the target and the salient distractor and thus may have received the highest suppression compared to the non-overlap condition. Moreover, previous studies showed that distractors with higher saliency levels produced stronger suppression signals^48,49^, which could explain why search impairment as observed primarily for the conditions with collinear distractors but not for the non-collinear distractors, due perhaps to collinear distractors being more salient than non-collinear distractors^18^. Further study is needed to confirm whether the signal suppression hypothesis applies to the collinear search impairment.

We argue that the long RTs observed in experiment 3 imply that the collinear search impairment may associate with spatial uncertainty of the target when it was embedded in the collinear distractor. The search display of the straight condition mimics those used in classical conditions^13^. However, RTs were much longer in a localization task (experiment 3) compared to a discrimination task (experiment 1). Interestingly, such prolonged responses were most obvious in the overlap condition (3534 ms for older and 759 ms for younger) than the non-overlap condition (1819 ms for older and 620 ms for younger). Since in the overlap condition target and the distractor were at the same sides of the display, such RT increment cannot be contributed to the response conflict from the opposite sides of the target and the distractor^29,37^. We argue that the search impairment induced by the collinear distractor may be linked to spatial uncertainty of the local target^50^, and such location-based interference in a localization task was aggravated with aging^42^. It is worth testing whether a target without location uncertainty can induce such search impairment. Such location-based attentional interference is also consistent with the signal suppression hypothesis because suppression is precisely observed only at the distractor location^48,51,52^.

Our findings are also consistent with recent studies on the search impairment via eye movement recording^53^. Hsiao et al.^53^, who recruited both younger and older adults to highlight individual differences, adopted eye movement analysis with the hidden Markov model to categorize individual eye movement traces. They found that older adults tended to have a more “dispersed” search pattern while younger adults tended to have a more “concentrated” search pattern. However, the search pattern did not correlate to the size of search impairment. Rather, the impairment was associated with the consistency of the first eye movement, which implies that the saccadic plan to non-saccadic goal locations (e.g., the collinear distractor) may contribute to search impairment. Therefore, our present and previous data consistently show that older adults did not qualitatively differ from younger adults in their processing stages; rather, older adults were more distracted and were slower.

Regarding attentional capture in the non-collinear distractor condition in experiment 1, our data are also consistent with previous observations that older adults are more distracted than younger adults. Although no significant attentional-capture effects were observed in younger adults, similar null results were also observed in other studies^13,22^, suggesting that attentional capture by non-collinear distractors is not a robust phenomenon. One possibility is that the non-collinear distractor was much larger than the target, making it inconsistent with attentional control settings of the target^10,54^. Another possibility is that the target was more salient^55^ in a homogeneous background (i.e., non-overlap) than in a heterogeneous background (i.e., overlap), canceling out the attentional-capture effect by the non-collinear distractor. Meanwhile, the observation that older adults exhibited such non-reliable attentional capture implies that they were more distracted than younger adults^37,41,56^.

One limitation to this study is that while we inferred that older adults may have a more declined grouping ability on collinear stimuli^4,5,7,8^, this was not directly measured. Nevertheless, the curved condition in Experiment 3 was like that used in Roudaia et al.^8^; the difference was that they used Gabors while we used bars. Alternatively, collinear grouping may be a unique perceptual grouping among other grouping laws; therefore, it is inevitable. Collinearity is processed very early in the early visual cortex^57^, while other groupings, such as the law of similarity, may be elicited later following object recognition^58,59^. A collinear contour can be integrated earlier than a similarity contour^60^, which could explain why search impairment can be observed within 40 ms of a display being presented^61^. Thus, collinear grouping may be a special case among grouping laws, in that critical processing of collinearity occurs earlier and is thus unavoidable in visual search even for older adults.

In summary, the data presented here indicate that aging does not mitigate but rather maintain collinear search impairment via prolonged processing speed in multiple underlying mechanisms. The findings do not support the idea that older adults are free from distraction of collinear grouping in the background. When given more time, older adults show the same attentional phenomenon as younger adults do. To help older adults perform efficient visual search, not only the salience of the target needs to be increased, but the distractors also need to be disorganized. In a real-world scenario, older adults may have difficulty in shopping in the supermarket if similar items are placed in an aligned manner in a long row. In view of this, smarter shelving, which aims at reducing collinear search impairment, may enhance shopping convenience for older adults.

## Supplementary Information

Supplementary Information.

## Data Availability

All data generated or analyzed during this study (the de-identified data) are available in the supplementary material.
