# Supplementary material for: Testing the effects of perceptual grouping on visual search in older adults
Source: Sci Rep. 2022 Nov 10;12:19231. doi: 10.1038/s41598-022-23139-3 (PMC9649716; doi:10.1038/s41598-022-23139-3)
Supplement: Supplementary file 1 — Supplementary Information. [file 41598_2022_23139_MOESM1_ESM.pdf]

Table S1: The ANOVA details for results of experiment 1

		ANOVA for response times			ANOVA for accuracy			ANOVA for RTz		
Source	df	MS	F	P	MS	F	P	MS	F	P
Age group (old vs young)	1	6012597	69.80	0	0.001	0.34	0.5662	0.013	1.16	0.2915
Error(Age)	28	86135			0.004			0.011		
Distractor type (collinear vs noncollinear)	1	71673	19.86	0.0001	0.034	17.40	0.0003	0.458	18.30	0.0002
Age X Distractor type	1	67383	18.67	0.0002	0.049	25.35	0	0.493	19.68	0.0001
Error (Age X Distractor type)	28	3609			0.002			0.025		
Target type (non-overlap vs overlap)	1	124124	41.18	0	0.052	25.12	0	1.26	59.15	0
Age X Target type	1	80809	26.81	0	0.027	12.82	0.0013	0.287	13.50	0.001
Error (Age X Target type)	28	3014			0.002			0.021		
Distractor type X Target type	1	246048	56.54	0	0.054	18.87	0.0002	2.313	63.10	0
Three-way interaction	1	177262	40.73	0	0.039	13.51	0.001	0.837	22.82	0.0001
Error (three-way interaction)	28	4352			0.003			0.037		

Note: df: degree of freedom, MS: mean square

Table S2: The ANOVA details for results of experiment 2

		ANOVA for response times			ANOVA for accuracy			ANOVA for RTz		
Source	df	MS	F	P	MS	F	P	MS	F	P
Age group (old vs young)	1	11449462	58.17	0	0.001	0.12	0.7327	0	0.00	1
Error(Age)	22	196812.9			0.005			0.021		
Target type (non-overlap vs overlap)	1	621225.5	18.16	0.0003	0.052	12.37	0.0019	3.994	41.30	0
Age X Target type	1	450603.8	13.18	0.0015	0.071	16.78	0.0005	1.048	10.84	0.0033
Error (Age X Target type)	22	34202.12			0.004			0.097		
Distractor length (3, 5, 9, 13 bars)	3	7166.585	4.43	0.0067	0.008	2.60	0.0597	0.063	2.43	0.073
Age X Distractor length	3	6152.704	3.81	0.014	0.009	3.02	0.0358	0.037	1.44	0.2649
Error (Age X Distractor length)	66	1616.603			0.003			0.026		
Distractor length X Target type	3	23278.71	9.80	0	0.005	1.59	0.2455	0.221	9.67	0
Three-way interaction	3	13856.27	5.83	0.0013	0.012	3.49	0.0204	0.027	1.18	0.3325
Error (three-way interaction)	66	2375.375			0.003			0.023		

Note: df: degree of freedom, MS: mean square

Table S3: The ANOVA details for the collinear search impairment effect of experiment 2

	ANOVA for response times				ANOVA for accuracy			ANOVA for RTz		
Source	df	MS	F	P	MS	F	P	MS	F	P
Effect for Age groups (old vs young)	1	901207.7	13.175	0.0015	0.142	16.78	0.0005	2.096	10.84	0.003
Error(Age)	22	68404.24			0.008			0.193		
Distractor length (3, 5, 9, 13 bars)	3	46557.43	9.8	0	0.011	1.59	0.2455	0.442	9.67	0
Age X Distractor length	3	27712.54	5.833	0.0013	0.023	3.49	0.0204	0.054	1.18	0.333
Error (Age X Distractor length)	66	4750.751			0.007			0.046		

Note: df: degree of freedom, MS: mean square

Table S4: The ANOVA details for results of experiment 3

		ANOVA for response times			ANOVA for accuracy			ANOVA for RTz		
Source	df	MS	F	P	MS	F	P	MS	F	P
Age group (old vs young)	1	123196132	34.88	0	0.271	5.03	0.0314	0.014	0.55	0.4642
Error(Age)	34	3532495			0.054			0.026		
Distractor type (curved vs straight)	1	583998	6.17	0.018	0.004	1.83	0.1876	0.032	1.14	0.2942
Age X Distractor type	1	676561	7.15	0.0114	0.023	11.42	0.0018	0.052	1.86	0.1848
Error (Age X Distractor type)	34	94623			0.002			0.028		
Target type (non-overlap vs overlap)	1	9584538	17.09	0.0002	0.012	0.47	0.4986	3.283	62.23	0
Age X Target type	1	6834057	12.19	0.0014	0.003	0.11	0.7474	0.056	1.06	0.3117
Error (Age X Target type)	34	560776			0.025			0.053		
Distractor type X Target type	1	6102773	29.29	0	0.013	5.24	0.0284	1.26	91.72	0
Three-way interaction	1	4465144	21.43	0.0001	0.005	2.08	0.1635	0.016	1.15	0.2914
Error (three-way interaction)	34	208325			0.002			0.014		

Note: df: degree of freedom, MS: mean square

Experiment1 Raw data

RT	Collinear diYtractor		Non-collinear diYtractor	
	non-overlap	overlap	non-overlap	overlap
Y1	488.2923077	488.6764706	496.4769231	507.5666667
Y10	503.4677419	564.5625	558.05	575.125
Y11	496.0441176	509.1515152	503	505.6129032
Y12	522.296875	531.1764706	542.1818182	522.1
Y13	506.8923077	491.0588235	492.9393939	498.6666667
Y14	469.328125	502.8387097	493.8064516	489.1666667
Y15	466.75	496.2758621	468.9090909	467.5625
Y2	564.9285714	571.8709677	565.265625	548.1428571
Y3	664.4603175	734.6060606	661.9117647	665.0857143
Y4	496	535.3214286	519.2463768	502.4666667
Y5	532.1969697	540.3333333	531.4923077	537.53125
Y6	527.796875	545.25	529.515625	545.0882353
Y7	504.1363636	513.3225806	501.9666667	500.34375
Y8	577.0655738	651.0357143	616.8392857	589.5625
Y9	479.0307692	514.9666667	500.2222222	508.7272727
O1	767.3692308	841.8888889	814.1029412	794.59375
O2	894.4179104	1014.821429	944.7846154	893.2424242
O3	1333.8	1684.689655	1476.542373	1420.275862
O4	670.1176471	803.0645161	730.8307692	692.5294118
O6	1020.552239	1575.375	1080.731343	1060.15625
O8	800.2205882	1195.545455	962.0769231	855.3125
O9	1049.290323	1251	1190.647059	1101.65625
O10	972.8125	1113.166667	1009.333333	995.1212121
O11	740.1267606	1095.208333	826.7936508	737.9090909
O12	1046.539683	1610.148148	1148.283582	987.3939394
O13	816.8088235	1019.15625	883.8412698	812.6060606
O14	716.0606061	921.6451613	699.921875	723
O15	856.1449275	1364.424242	890.1304348	892.0294118
O16	823.5373134	997.48	893.2575758	859.625
O17	785.5	1060.5	809.4090909	767.125

ACC	Collinear diYtractor		Non-collinear diYtractor	
	non-overlap	overlap	non-overlap	overlap
Y1	0.95588235	0.97142857	0.95588235	0.90909091
Y10	0.92537313	0.91428571	0.89552239	0.88888889
Y11	0.98550725	0.91666667	0.88059701	0.96875
Y12	0.96969697	1	0.97058824	0.85714286
Y13	0.95588235	0.97142857	0.94285714	0.94285714
Y14	0.92753623	0.91176471	0.91176471	0.83333333
Y15	0.88235294	0.85294118	0.94285714	0.94117647
Y2	0.8358209	0.96875	0.90140845	0.8
Y3	0.95454545	1	0.98550725	1
Y4	0.95774648	0.82352941	0.97183099	0.88235294
Y5	0.97058824	0.90909091	0.95588235	0.96969697
Y6	0.96969697	0.94117647	0.94117647	0.97142857
Y7	0.95652174	0.93939394	0.88235294	0.94117647
Y8	0.87142857	0.82352941	0.8358209	0.91428571
Y9	0.98484848	0.88235294	0.92647059	1
O1	0.94202899	0.79411765	1	0.96969697
O2	0.97101449	0.8	0.98484848	1
O3	0.95588235	0.82857143	0.89393939	0.85294118
O4	0.98550725	0.91176471	0.97014925	1
O6	0.98529412	0.70588235	0.95714286	1
O8	1	0.70967742	0.95588235	1
O9	0.95384615	0.91666667	1	0.94117647
O10	0.91428571	0.88235294	0.91304348	1
O11	1	0.75	0.96923077	1
O12	0.92647059	0.81818182	0.97101449	0.97058824
O13	0.97142857	0.91428571	0.96923077	1
O14	0.97058824	0.88571429	0.95522388	1
O15	1	0.94285714	0.98571429	0.97142857
O16	1	0.75757576	0.98507463	0.94117647
O17	1	0.70588235	1	0.96969697

zRT	Collinear diYtractor		Non-collinear diYtractor	
	non-overlap	overlap	non-overlap	overlap
Y1	-0.1734708	-0.1694443	-0.0876871	0.02854556
Y10	-0.4158363	0.02793164	-0.0193726	0.10465342
Y11	-0.216177	-0.0907348	-0.1496069	-0.1246005
Y12	-0.2351248	-0.1666635	-0.0818126	-0.2366427
Y13	-0.1111971	-0.2320434	-0.2176902	-0.1739778
Y14	-0.3025983	0.06206508	-0.0362243	-0.0867146
Y15	-0.1921118	0.04893758	-0.174485	-0.1854785
Y2	-0.1487981	-0.0962455	-0.1462467	-0.2758627
Y3	-0.1284134	0.08110157	-0.1360255	-0.1265454
Y4	-0.2994038	0.13918533	-0.040115	-0.227275
Y5	-0.174043	-0.1290472	-0.1779399	-0.1445433
Y6	-0.2010008	0.02236506	-0.1790042	0.02029479
Y7	-0.1383677	-0.0338191	-0.1630611	-0.1815315
Y8	-0.2349617	0.27177032	0.03750788	-0.1493516
Y9	-0.4021214	0.15689949	-0.072466	0.05983911
O1	-0.2932587	-0.0887265	-0.1649899	-0.2185362
O2	-0.3064184	0.14014883	-0.1196122	-0.3107782
O3	-0.3931868	0.22635881	-0.1411548	-0.2405013
O4	-0.4048534	0.43447506	-0.0215556	-0.2633621
O6	-0.4036879	0.67389472	-0.2868074	-0.3267686
O8	-0.5112408	0.66571727	-0.0293634	-0.3472216
O9	-0.2749346	0.07264459	-0.0313535	-0.1846994
O10	-0.2693617	-0.0067545	-0.20103	-0.2276214
O11	-0.4477561	1.02203571	-0.0890152	-0.4569357
O12	-0.3774014	0.7968224	-0.1654278	-0.5006259
O13	-0.2259804	0.03242061	-0.1403789	-0.2313474
O14	-0.2266868	0.38664941	-0.2748347	-0.205984
O15	-0.3064879	0.74338395	-0.2362894	-0.232367
O16	-0.3630745	0.30167127	-0.096629	-0.2251605
O17	-0.3092046	0.69802209	-0.2216342	-0.3765057

Experiment2 Raw data

RT	non-overlap				overlap			
	3	5	9	13	3	5	9	13
Y1	411.9333	416.6563	425.0313	420.4706	414.9412	424.3125	428.2667	409.4286
Y2	576.6129	598.7059	569.4242	577.9091	600.1875	620.3333	629.3529	636.375
Y3	490.3939	463.9355	452.5806	470	549.5714	497.9333	513.5882	523.625
Y4	484.1	458.6563	424.8333	481.2857	471.1333	467.75	455.0556	491.7143
Y5	455	456.0645	452.875	462.7273	454.1875	487.7333	474.6471	482.1875
Y6	423.9333	424.6452	424.129	407.68	434.5333	415.875	426.2857	461.625
Y7	553.9375	559.4848	551.6875	559.0667	594.2941	539.7059	584.0667	533.3333
Y8	642.4667	702.6667	683.3226	631.2941	713.9375	670	717.5556	756.5333
Y9	553.1034	554.6129	534.5455	568.3714	524.875	538.4444	555.4375	599.0625
Y10	591.2258	580.2121	572	576.931	576.375	595.375	593.4118	602
Y11	521.5313	498.7742	506.2121	477.125	473.8667	529.2222	532.125	479.4667
Y12	492.6364	501.7931	514.0588	498.3793	471.5714	514.5625	495.9444	503.1765
O1	875.8529	754.8125	792.9032	807.0882	709.875	803.7059	961.0588	953.4118
O2	719.0323	745.0571	758.6471	715.0625	796.4118	798.5	833.2143	865.5714
O3	732.8857	727.6286	720.2424	727.2571	776.875	720.125	807.4667	819.7273
O4	800.875	785.9688	800.375	765.0294	859.6471	791.8571	837.6667	845.9375
O5	1072.156	1056.176	1002.909	1003.667	1314.8	1272.667	1430.462	1351.214
O7	1091.938	830.8571	952.9355	901.0606	1011.5	1215.333	1237.143	1084.857
O8	940.4706	891.1667	938.7097	928.4333	910.625	914.7059	1008.353	980.4375
O9	904.1471	855.9688	820.9688	811.8387	1032.056	1214.2	1087.5	1347
O10	1003.667	997.4242	1049.771	1032.912	1485.5	1609.5	1747.846	1531.786
O11	1255.206	1124.029	1131.971	1073.903	1246.063	1311.5	1425.059	1494.923
O12	1028.194	1082.727	1084.706	996.0882	1491.5	1486.143	1713.467	1524.385
O14	881.2647	814.6129	819.8788	838.8485	937.3529	905.2667	966.9167	1087.571

ACC	non-overlap				overlap			
	3	5	9	13	3	5	9	13
Y1	0.857143	0.969697	0.914286	0.944444	1	0.941176	0.9375	0.823529
Y2	0.939394	0.971429	0.942857	0.970588	0.888889	1	0.944444	0.941176
Y3	0.942857	0.939394	0.939394	0.942857	0.823529	0.882353	1	0.941176
Y4	0.882353	0.914286	0.857143	0.823529	0.833333	0.941176	1	0.823529
Y5	1	0.939394	0.941176	0.942857	0.941176	0.9375	1	0.941176
Y6	0.909091	0.885714	0.885714	0.757576	0.882353	0.941176	0.823529	0.941176
Y7	0.914286	0.970588	0.969697	0.909091	0.944444	1	0.882353	0.882353
Y8	0.967742	0.942857	0.939394	0.971429	0.941176	0.944444	1	0.882353
Y9	0.90625	0.911765	0.970588	0.972222	0.941176	1	1	0.941176
Y10	0.911765	0.970588	0.914286	0.90625	0.941176	0.941176	1	1
Y11	0.914286	0.885714	0.916667	1	0.833333	1	0.941176	0.9375
Y12	1	0.90625	0.971429	0.878788	0.823529	0.888889	1	1
O1	0.971429	0.969697	0.96875	0.971429	0.888889	1	1	0.944444
O2	0.939394	1	0.971429	0.969697	1	1	0.875	0.875
O3	1	1	1	1	1	0.888889	0.882353	0.647059
O4	0.888889	0.969697	0.941176	0.971429	0.944444	0.823529	1	0.941176
O5	0.941176	0.971429	1	1	0.9375	1	0.866667	0.823529
O7	0.941176	0.933333	0.885714	0.916667	0.941176	1	0.777778	0.875
O8	0.971429	0.909091	0.911765	0.9375	0.941176	0.944444	0.944444	1
O9	0.971429	0.941176	0.914286	1	1	0.882353	0.75	0.777778
O10	1	0.970588	1	0.971429	0.941176	0.75	0.8125	0.823529
O11	1	0.971429	0.971429	0.96875	0.941176	0.941176	1	0.722222
O12	0.911765	1	1	0.944444	0.75	0.875	0.833333	0.8125
O14	1	0.911765	0.970588	0.942857	0.944444	0.9375	0.705882	0.823529

zRT	non-overlap				overlap			
	3	5	9	13	3	5	9	13
Y1	-0.2009	-0.11897	0.026311	-0.0528	-0.14873	0.013842	0.082436	-0.24435
Y2	-0.25487	-0.12938	-0.2957	-0.24751	-0.12097	-0.00654	0.044685	0.084569
Y3	-0.11892	-0.38798	-0.50345	-0.32631	0.482862	-0.04225	0.116945	0.21901
Y4	0.180645	-0.11748	-0.51378	0.14767	0.028714	-0.01093	-0.15967	0.269862
Y5	-0.20479	-0.18932	-0.23566	-0.09251	-0.21659	0.270827	0.080685	0.190246
Y6	-0.09957	-0.08806	-0.09641	-0.3623	0.071774	-0.22983	-0.06155	0.509702
Y7	-0.13587	-0.09416	-0.15278	-0.0973	0.167531	-0.24286	0.090642	-0.29077
Y8	-0.24487	0.01997	-0.06513	-0.29402	0.069554	-0.12374	0.085471	0.256947
Y9	-0.15227	-0.14223	-0.27572	-0.05071	-0.34005	-0.24978	-0.13675	0.153443
Y10	-0.06606	-0.12143	-0.16271	-0.13792	-0.14072	-0.04521	-0.05507	-0.0119
Y11	0.066444	-0.1546	-0.08236	-0.36489	-0.39654	0.141149	0.169345	-0.34215
Y12	-0.16385	-0.07082	0.053801	-0.10551	-0.37788	0.058918	-0.13024	-0.05677
O1	0.027529	-0.31167	-0.20492	-0.16517	-0.4376	-0.17465	0.266305	0.244876
O2	-0.39321	-0.24751	-0.17142	-0.41543	0.040006	0.051697	0.246048	0.427202
O3	-0.2259	-0.25706	-0.30084	-0.25926	0.034854	-0.30154	0.216189	0.288865
O4	-0.20263	-0.26969	-0.20488	-0.36389	0.061768	-0.2432	-0.03712	9.19E-05
O5	-0.25294	-0.2783	-0.36283	-0.36162	0.132093	0.065234	0.315628	0.189876
O7	-0.09304	-0.40949	-0.26152	-0.3244	-0.19054	0.056521	0.082955	-0.10162
O8	-0.16234	-0.30658	-0.16749	-0.19756	-0.24965	-0.23771	0.036238	-0.04543
O9	-0.27416	-0.38553	-0.46644	-0.48754	0.021511	0.442556	0.149676	0.749537
O10	-0.396	-0.40413	-0.33599	-0.35793	0.23121	0.392623	0.572712	0.291461
O11	-0.0602	-0.31303	-0.29772	-0.40964	-0.07782	0.048302	0.267173	0.401828
O12	-0.4859	-0.38422	-0.38054	-0.54575	0.377889	0.367901	0.791723	0.439199
O14	-0.18287	-0.38009	-0.36451	-0.30838	-0.01691	-0.11185	0.070564	0.427572

Experiment3 Raw data

RT	curve		straight	
	overlap	nonoverlap	overlap	nonoverlap
Y1	633.6364	612.8056	679.9429	557.8558
Y2	726.9459	727.066	760.9394	668.6262
Y3	701.1842	700.1604	923.4375	664.027
Y4	494.6471	499.1827	539.069	443.0283
Y5	712.1714	646	689.8438	632.4587
Y6	1005.139	953.7431	1269.886	808.6019
Y7	878.3143	771.7476	783.2973	760.8198
Y8	549.1563	526.4667	556.6486	475.4259
Y9	878.6667	909.1321	1069.559	871.78
Y10	761.2703	757.4757	760.3143	655.098
Y11	765.125	752.3905	945	627.1509
Y12	813.5152	742.8393	800.1176	630.1275
Y13	708.1892	688.6408	722.0968	608.9688
Y14	590.1143	574.0495	540.6765	505.6535
Y15	572.3611	630.8624	580.6216	541.787
Y16	624.6286	636.1376	656.9474	566.3585
Y17	688	642.7308	661.76	540.3789
Y18	670.6486	629.0909	730.4194	595.4952
O1	1880.514	1769.224	3037.184	1556.738
O2	1324.333	1093.131	1280	968.6733
O3	2152.6	1768.411	1800.486	1172.388
O4	2691.912	2829.869	4998.459	1799.566
O5	2066.281	1797.514	2853	1446.786
O6	760.1714	792.6389	783.6389	710.5588
O7	1184.949	1067.269	1419.194	827.6514
O8	.	1017.057	829.3333	905.6857
O9	4150.061	2101.019	4168.323	1621.714
O10	2260.459	1832.613	3593.176	1959.542
O11	2481.324	3595.811	3236.838	3069.857
O12	1864.276	1601.205	1923.645	1304.557
O13	1527.861	1439.262	2736.974	1539.514
O14	3284.528	3744.837	6032.829	2953.321
O15	4910.676	3492.288	7136.735	2934.613
O16	5261.808	3945.717	7064.348	1538.756
O17	2753.417	3394.205	4046.613	2636.375
O18	4552.412	4445.636	6677.4	3791.198

Accuracy	curve		straight	
	overlap	nonoverlap	overlap	nonoverlap
Y1	0.942857	0.964286	0.921053	0.920354
Y2	1	0.929825	0.891892	0.972727
Y3	1	0.954955	0.864865	0.991071
Y4	0.944444	0.936937	0.805556	0.921739
Y5	0.921053	0.936937	0.888889	0.964602
Y6	0.972973	0.95614	0.945946	0.936364
Y7	1	0.919643	0.973684	0.982301
Y8	0.888889	0.945946	0.948718	0.964286
Y9	0.972973	0.938053	0.894737	0.909091
Y10	0.973684	0.936364	0.972222	0.894737
Y11	0.864865	0.9375	0.710526	0.954955
Y12	0.891892	0.973913	0.918919	0.93578
Y13	0.948718	0.919643	0.861111	0.864865
Y14	0.921053	0.90991	0.871795	0.918182
Y15	1	0.973214	0.973684	0.964286
Y16	0.972222	0.973214	0.95	0.963636
Y17	0.846154	0.912281	0.657895	0.88785
Y18	1	0.973451	0.815789	0.954545
O1	0.948718	0.890909	0.974359	0.936364
O2	0.692308	0.75	0.72973	0.918182
O3	0.789474	0.863636	0.948718	0.765766
O4	0.894737	0.876106	1	0.9
O5	0.888889	0.973214	0.916667	0.982456
O6	0.921053	0.321429	0.972973	0.306306
O7	0.975	0.945455	0.947368	0.990909
O8	0	0.619469	0.078947	0.630631
O9	0.916667	0.964286	0.794872	0.945946
O10	1	0.972477	0.871795	0.946903
O11	1	0.938053	0.973684	0.954545
O12	0.763158	0.792793	0.794872	0.8
O13	1	0.963964	1	0.946903
O14	0.972973	0.890909	0.921053	0.938053
O15	0.894737	0.727273	0.871795	0.954955
O16	0.722222	0.807018	0.621622	0.810811
O17	0.685714	0.778761	0.794872	0.792793
O18	0.918919	0.955357	0.921053	0.90991

zRT	curve		straight	
	overlap	nonoverlap	overlap	nonoverlap
Y1	-0.03663	-0.12495	0.159705	-0.35792
Y2	-0.01608	-0.01541	0.172843	-0.34019
Y3	-0.14115	-0.14312	0.287123	-0.21275
Y4	-0.05517	-0.02815	0.209428	-0.36264
Y5	0.192334	-0.16966	0.070189	-0.24374
Y6	-0.0514	-0.11193	0.260395	-0.28287
Y7	0.213497	-0.16163	-0.12098	-0.2001
Y8	0.088115	-0.06935	0.140112	-0.42358
Y9	-0.24099	-0.18606	0.103183	-0.2534
Y10	-0.02557	-0.03759	-0.0286	-0.36176
Y11	-0.06171	-0.0942	0.397262	-0.41376
Y12	0.136579	-0.07037	0.097349	-0.40042
Y13	0.058007	-0.0225	0.115283	-0.35061
Y14	0.08282	-0.0007	-0.17421	-0.3563
Y15	-0.06945	-0.00752	-0.06071	-0.10182
Y16	-0.10116	-0.08425	-0.05367	-0.18678
Y17	0.129736	-0.09892	-0.00281	-0.61591
Y18	0.042092	-0.16782	0.344	-0.33752
O1	-0.13882	-0.18012	0.290427	-0.25897
O2	0.197229	-0.6425	0.03621	-1.09453
O3	0.20284	-0.03878	-0.0186	-0.41361
O4	-0.18413	-0.15756	0.260148	-0.35601
O5	-0.04541	-0.14842	0.256104	-0.28284
O6	-0.17262	-0.05248	-0.08578	-0.35619
O7	0.012834	-0.11202	0.261372	-0.36626
O8	.	-0.02631	-0.44535	-0.27491
O9	0.56747	-0.26273	0.574869	-0.45693
O10	-0.13703	-0.25423	0.22807	-0.21946
O11	-0.42683	-0.01819	-0.14981	-0.21104
O12	0.297162	-0.04797	0.375052	-0.43716
O13	-0.19376	-0.22863	0.282187	-0.18917
O14	-0.25839	-0.19159	0.140457	-0.30646
O15	0.127307	-0.20286	0.645482	-0.33268
O16	-0.04371	-0.16778	0.126213	-0.39468
O17	-0.20481	-0.03886	0.130091	-0.23512
O18	-0.16496	-0.19437	0.420199	-0.37458
